# Regulation of Tetraspanin CD63 in Chronic Myeloid Leukemia (CML): Single-Cell Analysis of Asymmetric Hematopoietic Stem Cell Division Genes

**DOI:** 10.3390/bioengineering12080830

**Published:** 2025-07-31

**Authors:** Christophe Desterke, Annelise Bennaceur-Griscelli, Ali G. Turhan

**Affiliations:** 1 Faculty of Medicine, University Paris Saclay, INSERM UMRS-1310, 94800 Villejuif, France; christophe.desterke@inserm.fr; 2 INSERM UMR-S-1310, University Paris Saclay, 94800 Villejuif, France; abenna@hotmail.fr; 3 Faculty of Medicine, University Paris Saclay, 94270 Le Kremlin Bicetre, France; 4 INGESTEM National iPSC Infrastructure, 94800 Villejuif, France; 5 CITHERA, Centre for IPSC Therapies, INSERM UMS-45, Genopole Campus, 91100 Evry, France

**Keywords:** asymmetric division, hematopoietic stem cell (HSC), CD63, chronic myeloid leukemia (CML)

## Abstract

(1) Background: Chronic myeloid leukemia (CML) is a myeloproliferative disorder driven by the BCR::ABL oncoprotein. During the chronic phase, Philadelphia chromosome-positive hematopoietic stem cells generate proliferative myeloid cells with various stages of maturation. Despite this expansion, leukemic stem cells (LSCs) retain self-renewal capacity via asymmetric cell divisions, sustaining the stem cell pool. Quiescent LSCs are known to be resistant to tyrosine kinase inhibitors (TKIs), potentially through BCR::ABL-independent signaling pathways. We hypothesize that dysregulation of genes governing asymmetric division in LSCs contributes to disease progression, and that their expression pattern may serve as a prognostic marker during the chronic phase of CML. (2) Methods: Genes related to asymmetric cell division in the context of hematopoietic stem cells were extracted from the PubMed database with the keyword “asymmetric hematopoietic stem cell”. The collected relative gene set was tested on two independent bulk transcriptome cohorts and the results were confirmed by single-cell RNA sequencing. (3) Results: The expression of genes involved in asymmetric hematopoietic stem cell division was found to discriminate disease phases during CML progression in the two independent transcriptome cohorts. Concordance between cohorts was observed on asymmetric molecules downregulated during blast crisis (BC) as compared to the chronic phase (CP). This downregulation during the BC phase was confirmed at single-cell level for SELL, CD63, NUMB, HK2, and LAMP2 genes. Single-cell analysis during the CP found that CD63 is associated with a poor prognosis phenotype, with the opposite prediction revealed by HK2 and NUMB expression. The single-cell trajectory reconstitution analysis in CP samples showed CD63 regulation highlighting a trajectory cluster implicating HSPB1, PIM2, ANXA5, LAMTOR1, CFL1, CD52, RAD52, MEIS1, and PDIA3, known to be implicated in hematopoietic malignancies. (4) Conclusion: Regulation of CD63, a tetraspanin involved in the asymmetric division of hematopoietic stem cells, was found to be associated with poor prognosis during CML progression and could be a potential new therapeutic target.

## 1. Introduction

Chronic myeloid leukemia (CML) is a malignancy characterized by the appearance of the leukemogenic oncoprotein breakpoint cluster region::Abelson murine leukemia viral oncogene homolog 1 (BCR::ABL), which is a molecular product of the Ph1 (Philadelphia) chromosome in hematopoietic stem cells (HSCs). This transforming effect in HSCs initiates a myeloproliferative process in the myeloid compartment, with the generation of myeloid cells with distinct stages of maturation in the bone marrow and the peripheral blood during the chronic phase [1]. The natural history of the disease includes the inexorable progression of the disease towards an aggressive blast crisis stage driven by genomic instability induced by BCR::ABL [2]. Extensive molecular profiling of the blastic phase has provided key insights into its pathogenesis [3]. The advent of treatment with tyrosine kinase inhibitors (TKIs) has significantly altered the natural course of chronic myeloid leukemia (CML), leading to marked improvements in patient survival and a substantial reduction in progression toward blast crisis. Despite these advances, a persistent challenge remains: the intrinsic resistance of primitive leukemic stem cells (LSCs) to TKIs, resulting in the long-term maintenance of minimal residual disease even in patients achieving deep molecular responses [4,5]. The persistence of CML LSC resistance to TKIs could be independent of the activity of the oncoprotein BCR::ABL, a phenomenon recognized as oncogene independence [6,7,8]. Deregulation of stem cell pathways such as perturbation tumor protein p53 (TP53), MYC proto-oncogene, bHLH transcription factor (MYC) networks [9], and Wingless-related Integration Site (WNT) signaling activation [10], or interaction between MYC and transcription factor 7-like 2 (TCF7L2) at the chromatin level [11], have also been described during CML blast crisis. Such disruptions in key regulatory pathways and factors governing leukemic stem cell biology intensify cellular stress within the pathological hematopoietic stem cell compartment, contributing to therapeutic resistance and altering cell fate decisions throughout CML progression.

To avoid proliferative stress and exhaustion, normal hematopoietic stem cells (HSCs) spend most of their lives outside of the cell cycle in a reversible quiescent stage and enter into the cell cycle every few months. Rather than undergoing continuous division, HSCs spend the majority of their lifespan in a reversible quiescent state, a non-cycling phase that minimizes replicative stress, prevents telomere erosion, and safeguards the integrity of the stem cell pool [12]. To take decisions between quiescence and proliferation, asymmetric cell divisions (ACD) are necessary for HSCs generating daughter cells engaged in differentiation and the persistent self-renewal potential of HSCs [13]. In primitive hematopoietic compartments, 20–30% of hematopoietic cells produce daughter cells that differ from each other in respect of their proliferation kinetics and/or their adopted cell fates [14,15,16]. During the asymmetric processes of hematopoietic stem cells, some molecules have been found to be differentially segregated between daughter HSCs and they belong to distinct organelles like endosomes (clusters of differentiations 71, 53,and 63) (CD71, CD53, and CD63) [17], mitochondria (DNML1: Dynamin-1-like protein) [18] or lysosomes supporting asymmetric organelle inheritance during hematopoietic cell fate decisions [19]. Some molecules known to be implicated in the polarity of cells such as NUMB endocytic adaptor protein (NUMB) [20] and cell division cycle 42 (CDC42) [21,22] were have also been described as associated with the asymmetric inheritance of daughter HSCs. Musashi (MSI) is a known repressor of NUMB translation and consequently an activator of Notch signaling [23]. The Musashi-2–NUMB axis has been identified as a potential regulatory pathway involved in hematopoietic differentiation and leukemic progression of myeloid malignancies [24]. In the context of acute myeloid leukemia (AML), the loss of CDC42 disrupts cell polarity and division asymmetry, leading to the alteration of leukemia-initiating cell fate in differentiation therapy [25]. Mitochondrial asymmetric inheritance was found associated with distinct metabolisms in HSCs [19], and carbohydrate metabolic markers (hexokinase 2 (HK2) and GLUT1 alias “SLC2A1” for Solute Carrier Family 2 Member 1) were found to be asymmetrically distributed between HSCs and daughter cells after in vivo interferon alpha administration [26]. In acute myeloid leukemia (AML), nuclear HK2 interacts with chromatin-regulating proteins to enhance chromatin accessibility at loci associated with leukemic stem cell signatures and DNA repair pathways. Overexpression of nuclear HK2 leads to a reduction in double-strand DNA breaks and promotes chemoresistance, highlighting a non-canonical mechanism by which leukemic stem cells may evade the cytotoxic effects of DNA-damaging agents [27]. GLUT1 (SLC2A1) is a key glucose transporter that supports glycolytic flux. In leukemic stem cells, rapamycin derivatives (RDs) reduce GLUT1 mRNA expression by inhibiting mTORC1, thereby limiting glucose uptake [28]. Given the dual involvement of certain molecules in both asymmetric hematopoietic stem cell division and the pathogenesis of transformed myeloid malignancies, this study aimed to comprehensively examine their expression dynamics during chronic myeloid leukemia (CML) progression.

In this study, a curated set of genes linked to asymmetric division in hematopoietic stem cells was identified from the recent literature. Their expression dynamics were examined across two independent transcriptomic cohorts of chronic myeloid leukemia (CML), each encompassing distinct disease phases. The analysis revealed that asymmetric division-related markers (SELL, CD63, NUMB, HK2, and LAMP2) effectively distinguished the chronic phase from the blast crisis phase, with consistent downregulation observed during disease progression, particularly in the blast crisis phase. Among these, CD63 emerged as a key marker, showing significant repression not only in bulk transcriptomes but also at the single-cell level within the CD34^+^CD38^−^ compartment. Furthermore, a prognostic asymmetry risk score, derived from single-cell data during the chronic phase, was found to correlate with patient outcomes, underscoring CD63’s central role in linking loss of asymmetric division properties with poor TKI response and unfavorable prognosis.

## 2. Materials and Methods

### 2.1. Public Datasets

#### 2.1.1. Training Cohort of Transcriptome (GSE4170)

In the classical cohort reported, CML mononuclear cells from patients in chronic phases (42 cases) and blast phases (32 cases) were investigated with the transcriptome microarray technology the Rosetta/Merck Human 25k v2.2.1 microarray [3]. A microarray normalized matrix downloaded on the Gene Expression Omnibus [29] was used for downstream analyses.

#### 2.1.2. Validation Cohort of RNA Sequencing (GSE100026)

Total RNA samples from peripheral blood mononuclear cells of CML patients in the chronic phase (5 samples) and blast crisis phase (5 samples), and also from healthy donors (5 samples), were processed by the Illumina protocol to perform RNA sequencing on Illumina NextSeq 500 technology [30]. After sequencing, reads were aligned to the hg19 whole genome using tophat2 [31]. Reads per kilobase of exon model per million mapped reads (RPKM) were normalized by edgeR [32] for downstream analyses.

#### 2.1.3. Single-Cell Transcriptome of CML Progenitors (GSE76312)

Single-cell transcriptomes performed on Lin-CD34+CD38- from CML patients in the chronic phase and during blast crisis [33] were downloaded on the Gene Expression Omnibus website [29] with corresponding metafile information to build a single-cell Seurat object [34]. This single-cell dataset processed primitive cells obtained from 16 patients in the chronic phase and 5 patients in blast crisis. Blast crisis patients were characterized with lymphoid blast crisis in two cases, with myeloid blast crisis in one case, and with pre-blastic stage at diagnosis in two cases. Concerning cells at the diagnosis stage from the chronic phase, sixteen patients were investigated. Accordingly, a if major molecular response (MMR: BCR::ABL transcript level < 0.1%) was achieved under the first line of tyrosine kinase therapy (Imatinib, Bosutinib, Nilotinib, or Dasatinib), chronic phase patients were classified into two groups: good responders (achieved MMR, n = 11) and poor responders (not achieved MMR, n = 5). There was no significant difference between the two groups during the chronic phase in terms of type of tyrosine kinase inhibitor employed (chi square test *p*-value = 0.1730), but also in percentages of BCR::ABL positive cells analysed (means (standard deviation)): responders: 60.9% (+/− 25.9%), non-responders (58.2% (43.1%)) (2-sided *t* test *p*-value = 0.8744). All analyzed patients presented classical BCR::ABL t (9–22) except one patient in the chronic phase who presented additional abnormality on chromosome 15. This patient was classed as a good responder after Nilotinib tyrosine kinase inhibitor therapy.

### 2.2. Analyses

#### 2.2.1. Screening of the Genes in the Literature Related to the Term “Asymmetric Hematopoietic Stem Cell”

The PubMed database [35] was queried on 24 March 2025 with the term “asymmetric hematopoietic stem cell”. Timeline analysis of article counts was exported as a csv file. The last seven years of full-text reviews (from 2018 to 2025) (Supplemental Table S1) were individually read to extract gene symbols connected to the terminology. When full-text manuscripts were present in the PubMed central database, text mining work was conducted with the “Pubtator3” server application: (https://www.ncbi.nlm.nih.gov/research/pubtator3/, accessed on 24 February 2025) [36]. Statistics were summarized as bar plots with ggplot2 R-package version 3.4.0 [37].

#### 2.2.2. Transcriptome Analyses

Transcriptome analyses were performed in R software environment version 4.2.1. Normalized matrices were reduced to the quantification of identified asymmetric hematopoietic stem cell-related genes. Unsupervised clustering associated with expression heatmaps was conducted with Euclidean distances and the “Ward.D2” method implemented in pheatmap R-package version 1.0.12. Principal component analysis performed on asymmetric hematopoietic stem cell-related genes was conducted with the “prcomp” R base function and plotted with autoplot from the ggfortify R-package 0.4.15. Supervised machine learning on asymmetric hematopoietic stem cell-related genes was conducted by a leave-one-out cross-validation algorithm implemented in pamr R-package version 1.56.1 [38].

#### 2.2.3. Single-Cell Asymmetric Hematopoietic Stem Cell Risk Score

A single-cell RNA sequencing transcriptome from a GSE76312 experiment was integrated into a Seurat single-cell object with metafile phenotype information (Seurat R-package version 4.3.0) [34]. Single-cell objects were filtrated for cells with a minimal expression of one hundred genes and genes with minimal expression in three cells. Quality assessment was checked with scatterplots between numbers of features and numbers of genes by cells followed by quality filtration. Single-cell RNA sequencing was normalized and scaled before dimension reduction with principal component analysis on fifty components. A second-dimension reduction by t-distributed stochastic neighbor embedding (tSNE) was operated on thirty dimensions of principal component analysis. Dimplot, dotplot and violinplot single-cell visualizations were performed on cells of interest. An asymmetric hematopoietic stem cell risk score was computed with the Seurat function “AddModuleScore” based on expression of the five asymmetric genes: selectin L (SELL), lysosomal-associated membrane protein 2 (LAMP2), cluster of differentiation 63 (tetraspanin CD63), NUMB, and HK2. With cells from chronic phases, the risk score was integrated in a logistic multi-variable model with poor prognosis as the binomial outcome. A multi-variable model was built and calibrated by bootstrapping with rms R-package version 6.4-1. A corresponding nomogram was drawn with regplot R-package version 1.1.

#### 2.2.4. Correlation of Single-Cell Artificial Neural Network with Prognosis and Outcome

An artificial neural network built with input chronic phase single-cell expression of SELL, LAMP2, CD63, NUMB, and HK2 and prognosis status was evaluated. The model was tuned with caret R-package version 6.0-93 by cross validation with the “nnet” algorithm in package version 7.3-17. Variable importance was estimated with Garson’s algorithm implemented in NeuralNetTools R package version 1.5.3 [39]. Lek’s profiles, which explore relationships between outcomes and continuous predictors, were computed on quantiles for SELL, LAMP2, CD63, NUMB, and HK2 single-cell expression. Black box opening of neural network models [40] was conducted by exploring single-cell explanations of 25 random cells by local interpretable model-agnostic explanations (LIME) in R-package lime version 0.5.3, and partial dependency between HK2 and NUMB single-cell expression was explored with pdp R-package version 0.8.1.

#### 2.2.5. Single-Cell Trajectory

CML-CP cells at diagnosis were used to build cell trajectories with monocle R-package version 2.22.0 [41]. Pseudotime differential expression analysis was performed on good versus poor prognosis cell patient phenotypes. Pseudotime cell trajectories were reconstituted with the “ddrtree” algorithm [42] and pseudotime expression heatmaps were drawn on the most significant genes found on the trajectories. Pseudotime expression plots were performed for relevant markers found following CD63 during pseudotime trajectories. Functional enrichment on the DisGeNet [43] and Gene Ontology [44] databases was performed for genes stratified in CD63 pseudotime clusters with the Toppgene server [45]. Functional enrichment networks were drawn with Cytoscape software version 3.9.1 [46].

## 3. Results

### 3.1. Identification of Asymmetric Hematopoietic Stem Cell-Related Genes in CML

**Figure 1:**
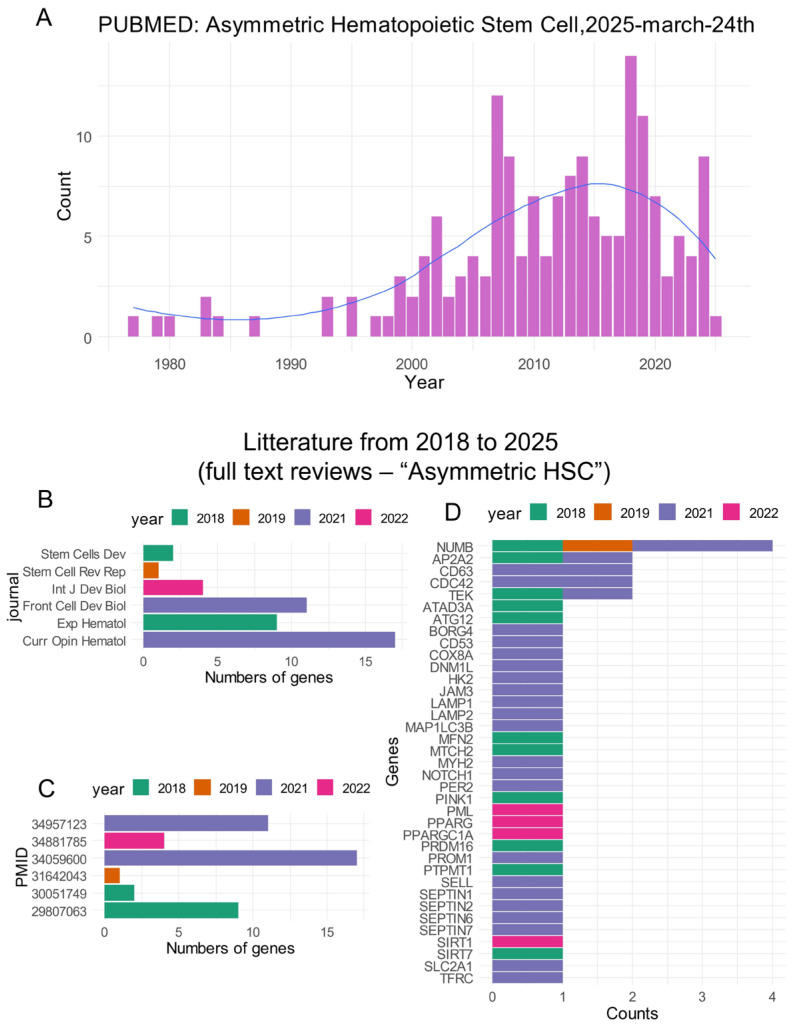
Asymmetric hematopoietic stem cell-related literature: (**A**) Timeline bar plot of article counts found in the PubMed database for the query “asymmetric hematopoietic stem cell”; (**B**) counts of asymmetric hematopoietic stem cell-related genes highlighted in the reviews during the last seven years (bar plot by journal); (**C**) same as (**B**) with bar plot by PMID numbers; and (**D**) bar plot of gene counts identified as associated with asymmetric hematopoietic stem cell literature.

A PubMed database query for the term “Asymmetric hematopoietic stem cell” performed on 24 March 2025 showed a progressively greater interest in this research field in the twenty last years (Figure 1A). By analyzing review articles from 2018 to 2025, we were able to identify six full text articles with annotated gene relations on the “Pubtator3” text-mining server. These review articles were published in distinct journals (Figure 1B) and have distinct yields of gene identification. The year 2021 was found to be particularly productive (Figure 1C). Among genes identified as being associated with the “Asymmetric hematopoietic stem cell” phenotype (Supplemental Table S1), some genes cited by distinct articles included NUMB (NUMB endocytic adaptor protein), AP2A2 (adaptor related protein complex 2 subunit alpha 2), CD63 (cluster of differentiation 63), CDC42 (cell division cycle 42), and TEK (TEK receptor tyrosine kinase) (Figure 1D).

### 3.2. Expression of Asymmetric Hematopoietic Stem Cell-Related Genes Discriminate Disease Phases During CML Progression

**Figure 2:**
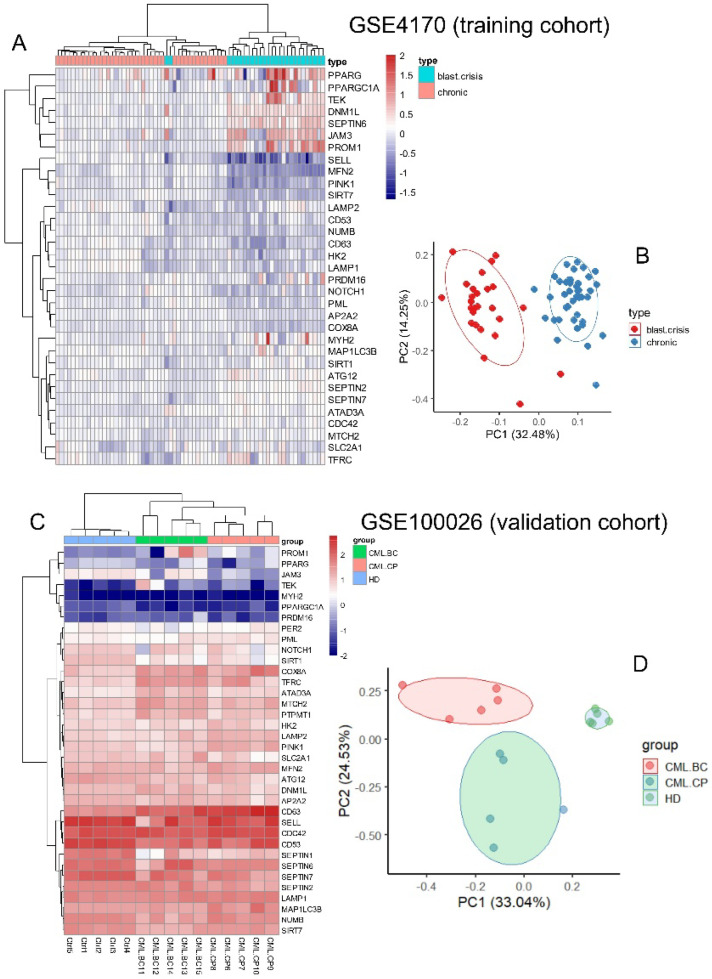
Expression of asymmetric hematopoietic stem cell-related genes discriminates disease phases during CML progression: (**A**) heatmap with unsupervised clustering based on expression of asymmetric hematopoietic stem cell-related genes on training cohort GSE4170; (**B**) principal component analysis conducted with expression of asymmetric hematopoietic stem cell-related genes on training cohort GSE4170; (**C**) heatmap with unsupervised clustering based on expression of asymmetric hematopoietic stem cell-related genes on validation cohort GSE100026; (**D**) principal component analysis conducted with expression of asymmetric hematopoietic stem cell-related genes on validation cohort GSE100026.

Expression of the asymmetric hematopoietic stem cell-related genes (Supplemental Table S1) was investigated in two independent cohorts of CML transcriptome GSE4170 and GSE10026 by two distinct unsupervised analyses: hierarchical clustering on Euclidean distances and principal component analysis. Clustering on the GSE4170 training cohort allowed us to discriminate the majority of the samples between chronic phases and blast crisis phases in two distinct clusters (Figure 2A). On the heatmap it could be observed that there was clear upregulation of peroxisome proliferator-activated receptor gamma (PPARG), PPARG coactivator 1 alpha (PPARGC1A), TEK, DNML1, septin 6 (SEPTIN6), junctional adhesion molecule 3 (JAM3), and prominin 1 (PROM1), and clear downregulation of SELL, mitofusin 2 (MFN2), PTEN-induced kinase 1 (PINK1), sirtuin 7 (SIRT7), and CD63 during blast crisis as compared to during the chronic phase (Figure 2A). Supervised machine learning performed on the training cohort confirmed the major importance of DNML1, SEPTIN6, JAM3, PROM1, and TEK upregulation and downregulation of MFN2, SELL, PINK1, SIRT7, and CD63 during blast crisis (Supplemental Figure S1A). The disease phase stratification of the training cohort was confirmed by principal component analysis, especially on the first principal component axis weighted at 32 percent of the variance (Figure 2B). The same analyses were applied on validation cohort GSE100026, which also comprised CML and healthy donor samples. Asymmetric hematopoietic stem cell-related genes also well stratified the sample phenotype by unsupervised clustering (Figure 2C). Supervised machine learning allowed us to uncover important upregulation of JAM3, ATPase family AAA domain containing 3A (ATAD3A), TEK, PR/SET domain 16 (PRDM16), and septin 2 (SEPTIN2), along with important downregulation of PINK1, CD63, SIRT7, NUMB, and LAMP2 during blast crisis versus chronic phase samples of the GSE100026 validation cohort (Supplemental Figure S1B). Disease phase stratification of the validation was confirmed by unsupervised principal component analysis (Figure 2D), especially on the first principal axis weighted at 33 percent of the variance. These results suggest that regulation of the asymmetric hematopoietic stem cell-related genes allows discrimination of hematopoietic cells from the distinct phase of the CML with reproducibility between two independent cohorts.

**Figure 3:**
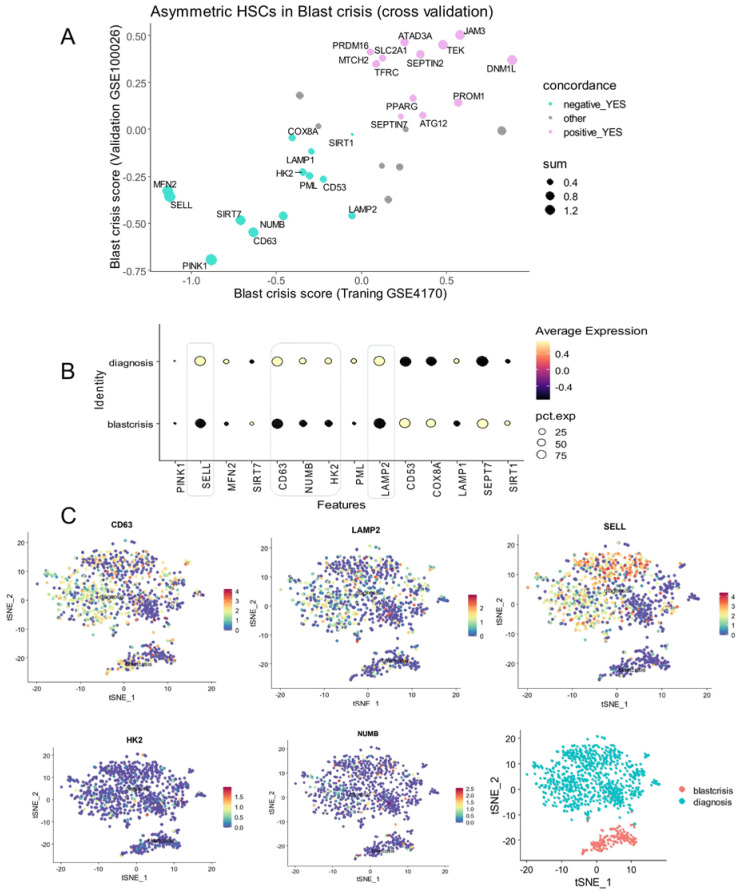
Repression concordance of asymmetry related genes during CML blast crisis: (**A**) scatterplot of predictive scores for asymmetry-related genes between training and validation cohorts; (**B**) single-cell expression of repressed asymmetry-related genes during CML blast crisis; (**C**) chronic and blast crisis phase expression of representative asymmetric markers repressed during blast crisis (tSNE dimension reduction).

The concordance analysis of the machine learning predictive scores between the two independent cohorts showed that the majority of the asymmetric hematopoietic stem cell-related genes were found to be regulated in the same sense during the progression of the disease in the two independent cohorts (Supplemental Table S2 and Figure S3A). To confirm regulation of asymmetric genes during phase progression of the disease, genes with concordant regulations (Supplemental Table S2) were investigated in a single-cell transcriptome of CML CD34+CD38− from GSE76312 dataset (28) between the chronic and blast crisis phases. These analyses revealed that only some downregulated asymmetric division genes during blast crisis were also confirmed to be downregulated at single-cell level. These genes include SELL, CD63, NUMB, HK2, and LAMP2 (Figure 3B) with high expression of SELL, CD63, and LAMP2 during the chronic phase and more moderate expression for HK2 and NUMB (Figure 3C).

**Figure 4:**
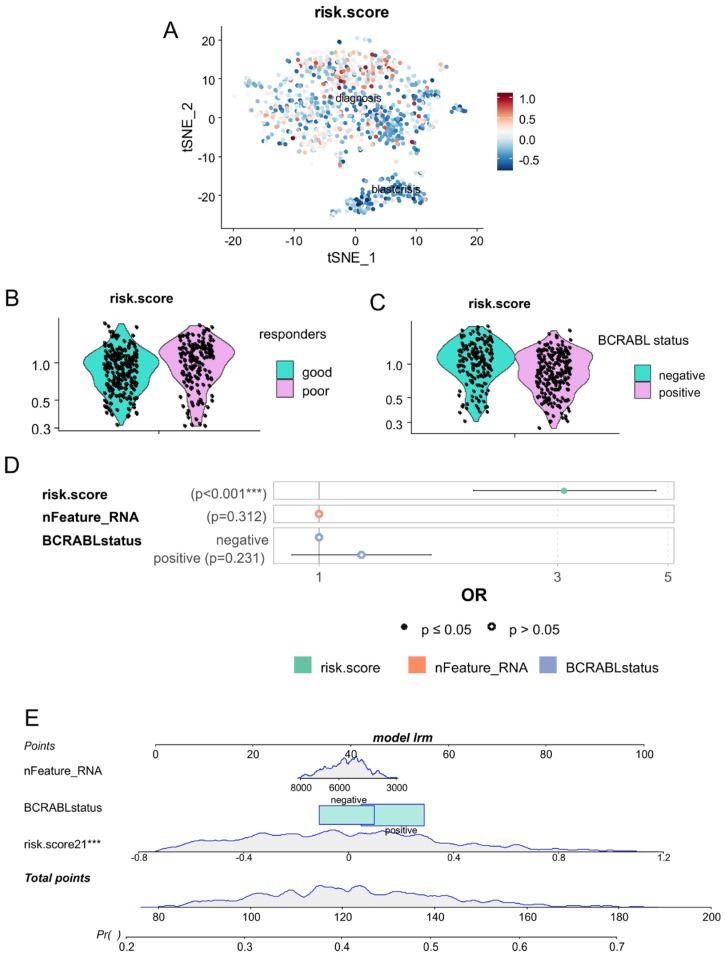
Single-cell risk score based on expression of asymmetric hematopoietic stem cell genes is an independent marker of CML blast crisis: (**A**) single-cell computed risk score (CD63, LAMP2, SELL, HK2, and NUMB) quantified during chronic and blast crisis phases (tSNE dimension reduction); (**B**) Violin plot of risk score for chronic phase cells stratified on prognosis of CML patients (good and poor responders); (**C**) Violin plot of risk score for chronic phase cells stratified on single-cell detection of BCR::ABL; (**D**) multi-variable logistic model (outcome: blast crisis versus chronic phase status of the cells) testing independence of single-cell risk score as compared to number of genes quantified in each cell and detection of BCR::ABL (***: *p*-value <= 0.01); and (**E**) nomogram of the single-cell multi-variable logistic model.

Based on the expression of these five asymmetric genes preferentially expressed in cells during the chronic phase, we have established an asymmetry-related risk score for each cell from blast crisis and chronic phases. This asymmetric risk score was confirmed to be higher in some cells in the chronic phase (Figure 4A). Focused on cells in the chronic phase, the asymmetry risk score was found to be higher in cells from patients with poorer prognosis (Figure 4B), but also higher in cells which were characterized as negative for BCR::ABL fusion genes (Figure 4C). In a multi-variable logistic regression model with poor prognosis as an outcome, the asymmetric risk score in the chronic phase cells was found with significant association independently of the number of features quantified by cells and of the BCR::ABL status of the cells (multi-variable *p*-value < 0.001, Figure 4D).

### 3.3. The CP-CML Single-Cell Trajectory Correlated to the CD63 Expression Profiles of Hematopoietic Malignancies and TKI Response

**Figure 5:**
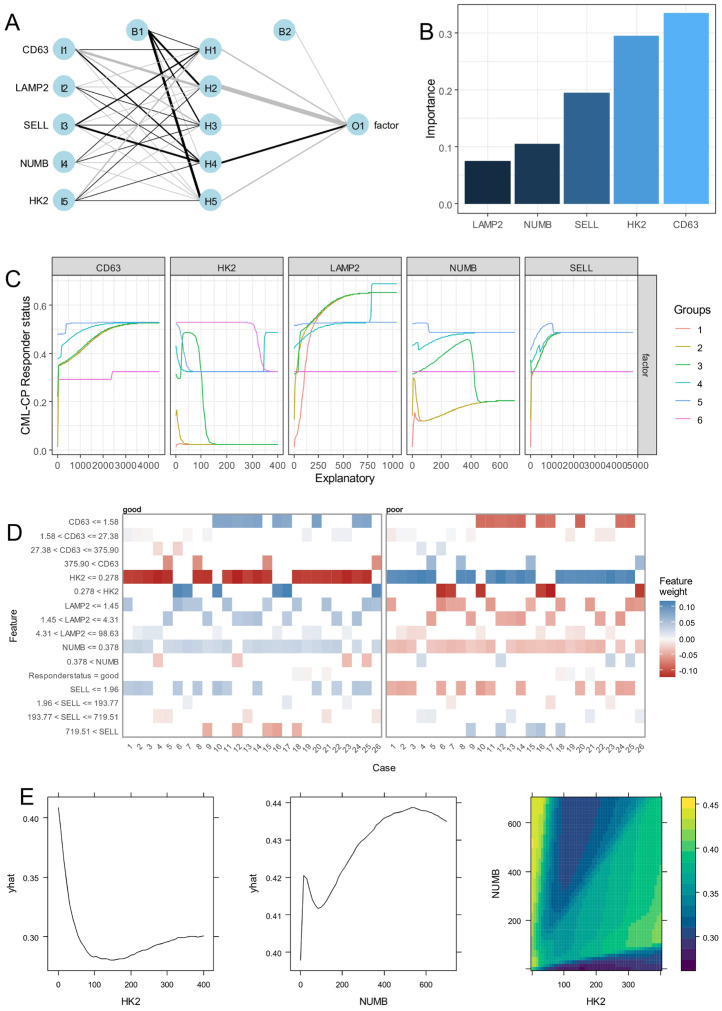
CD63 single-cell expression during the chronic phase is the best asymmetric marker predictive of the CML poor prognosis: (**A**) chronic phase single-cell neural network based on expression of asymmetric hematopoietic stem cell markers with poor prognosis as outcome; (**B**) variable Garson’s importance determined on poor prognosis neural network. (**C**) Lek’s profiles for continuous variables introduced in single-cell poor prognosis neural network; (**D**) Single-cell explanation of poor prognosis neural network model; (**E**) HK2 or/and NUMB poor prognosis predict probability in single-cell neural network model (yhat: probability lines).

Based on the expression of the five asymmetric markers—SELL, CD63, NUMB, HK2, and LAMP2—found repressed during blast crisis, a neural network was built to understand their single-cell expression heterogeneity during the CP according to the good or poor responses of the patients (Figure 5A). Black box explanation of the neural network highlighted higher importance of CD63, followed by HK2, SELL, NUMB, and LAMP2 in their expression to predict chronic phase poor prognosis (Figure 5B). Lek’s profile segmentation revealed the distinct concordance of the five markers according to the probability prediction of poor TKI response status during the chronic phase (Figure 5C). As can be seen in the figure, a progressive increase in CD63 and LAMP2 with the majority of the cell groups, a heterogenous reduction in probability of HK2 according to cell groups, a distinct concordance in probability of NUMB expression according to cell groups, and a probability increase for SELL expression were found to be associated with poor TKI response (Figure 5C). Single-cell feature weight highlighted the importance of CD63 expression at a threshold of 1.58, HK2 expression at that of 0.278 and NUMB expression at that of 0.378 in the decision of the neural network (Figure 5D). The partial probability of prediction was found to be opposite according to the expression level of HK2 and NUMB and their combinations defined waves of decisions in the network (Figure 5E). These results suggested strongly the main role of CD63 expression in predicting poor therapy response during the chronic phase followed by the opposite regulation of HK2 and NUMB.

**Figure 6:**
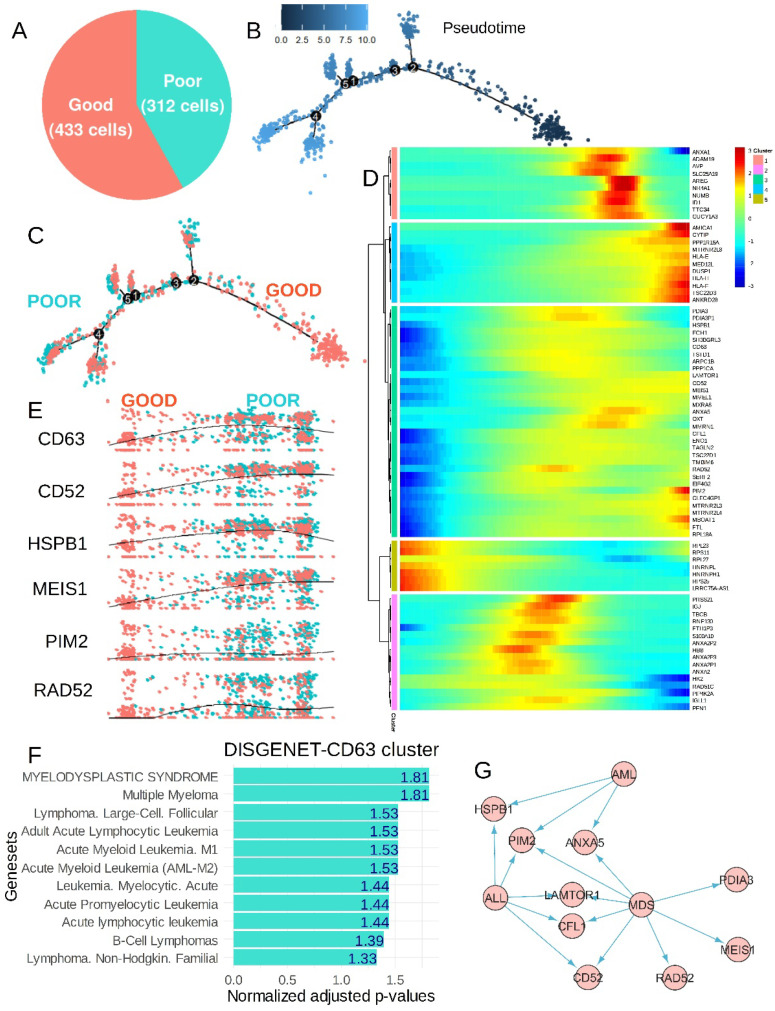
Poor prognosis CML chronic phase single-cell trajectory-associated CD63 expression heterogeneity-to-expression profile of several hemopathies: (**A**) proportion of good and poor prognosis CML chronic phase cells used to build trajectory; (**B**) pseudotime tree computed on prognosis of CML chronic phase cells, the number scale represents pseudotime quantification; (**C**) cell prognosis stratification on computed pseudotime tree during CML chronic phase; (**D**) pseudotime expression heatmap of significant genes found on cell prognosis trajectory, the number scale represents pseudotime quantification; (**E**) pseudotime expression of some representative markers which followed expression of CD63 during prognosis trajectory of CML chronic phase; (**F**) functional enrichment performed on DisGeNet disease database with genes from the CD63 cluster (negative log10 *p*-value numbers are indicated in bars); and (**G**) signature of hemopathies (MDS—myelodysplasia; ALL—acute lymphoid leukemia; AML—acute myeloid leukemia) among genes found in the CD63 trajectory cluster (arrows = interactions).

According to the TKI response status of the patients (associated with good/poor prognosis) a single-cell pseudotime trajectory was reconstituted for the CP-CML cells (Figure 6A,B). The inference of the prognosis status on the cell trajectory stratified the majority of poor prognosis cells (poor TKI response) on the left and the majority with good prognosis on the right of the trajectory (Figure 6C). Expression heterogeneity of CD63 was found to be highly significant on this cell trajectory (*p*-value = 3.755 × 10^−7^, Supplemental Table S3) and defined a green trajectory cluster (Figure 6D) comprising markers like cluster of differentiation 52 (CD52), heat shock protein family B (small) member 1 (HSPB1), Meis homeobox 1 (MEIS1), Pim-2 proto-oncogene, serine/threonine kinase (PIM2), RAD52 homolog, and DNA repair protein (RAD52) following CD63 expression (Figure 6E). On single-cell prognosis trajectory, HK2 expression heterogeneity (pink cluster, Figure 6D) was confirmed associated with a distinct CD63 cluster (green cluster). In the HK2 pink cluster, expression of RAD51 paralog C (RAD51C) and S100 calcium-binding protein A10 (S100A10) were found concordant with HK2 expression (Supplemental Figure S2D). NUMB expression heterogeneity (salmon cluster, Figure 6D) was confirmed associated with a distinct CD63 cluster (green cluster). In NUMB salmon cluster expression of nuclear receptor subfamily 4 group A member 1 (NR4A1) and inhibitor of DNA binding 1, HLH proteins (ID1) were found concordant with NUMB expression (Supplemental Figure S2E). Functional enrichment performed on markers of the CD63 green cluster with the Gene Ontology Biological Process database highlighted major implications of these molecules in negative regulation of apoptotic processes (Supplemental Figure S2A). The functional enrichment network showed the implication of MT-RNR2-like 4 (pseudogene) (MTRNR2L4), PIM2, annexin A5 (ANXA5), transmembrane BAX inhibitor motif-containing 6 (TMBIM6), enolase 1 (ENO1), heat shock protein family B (small) member 1 (HSPB1), MT-RNR2 like 3 (pseudogene) (MTRNR2L3), TSC22 domain family member 1 (TSC22D1), and CFL1 in negative regulation of apoptosis and ENO1, transmembrane BAX inhibitor motif-containing 6 (TMBIM6) could be hypoxia induced (Supplemental Figure S2B). In the CD63 green cluster some primary lysosomal markers such as late endosomal/lysosomal adaptor, MAPK and MTOR activator 1 (LAMTOR1), and ferritin light chain (FTL) were found associated (Supplemental Figure S2C). Functional enrichment performed on markers of the CD63 green cluster with the DisGeNet disease database highlighted significant enrichment in signatures of acute/chronic-lymhoid/myeloid blood disorders (Figure 6F). PIM2 was linked to acute myeloid leukemia (AML), acute lymphoid leukemia (ALL), and myelodysplasia (MDS), LAMTOR1, cofilin 1 (CFL1), and cluster of differentiation 52 (CD52) were shared between ALL and MDS phenotypes, ANXA5 was shared between MDS and AML phenotypes, and protein disulfide isomerase family A member 3 (PDIA3), MEIS1, and RAD52 were specifically associated with the MDS phenotype (Figure 6G).

## 4. Discussion

Despite the major progress obtained in CML therapy using TKIs, the resistance of leukemic stem cell tyrosine kinase inhibitors leading to their long-term resistance is a now well-established concept [4,5]. Asymmetric cell division, whereby two daughter cells adopt distinct identities, is a key process in generating cellular diversity in multicellular organisms. [47]. This property characterizes stem cells, which allows one daughter cell to retain stemness properties, whereas the other daughter cell becomes more differentiated [48]. During human hematopoiesis, the property of asymmetric organelle inheritance has also been demonstrated to participate in asymmetric processes during daughter cell division of this primitive hematopoietic compartment [19]. In the present study, genes associated with asymmetric division of hematopoietic stem cells were identified through a targeted literature review (Figure 1) and subsequently analyzed across two independent bulk transcriptomic cohorts of chronic myeloid leukemia (CML) patients at distinct stages of disease progression. The expression profiles of these markers consistently distinguished chronic phases from blast crisis phases, highlighting a reproducible transcriptomic signature of asymmetric stem cell regulation during CML evolution. The regulation concordance between the two distinct cohorts was principally observed in asymmetric cell division molecules downregulated during blast crisis as compared to the chronic phase, suggesting fewer asymmetric properties during the advanced phase of the disease. It is well established that CML leukemic stem cells reside in primitive CD34+/CD38− phenotype fractions as normal hematopoietic stem cells [49,50]. The blast crisis downregulation of asymmetric markers was confirmed at the single-cell level in the CD34+/CD38− compartment for SELL, CD63, NUMB, HK2, and LAMP2 expression. During the CP, single-cell neural network cells highlighted the major importance of CD63 regulation to elucidate poor TKI response/poor prognosis phenotypes, with the opposite prediction for HK2 and NUMB. CD63 is a membrane-bound protein composed of four transmembrane segments. While it is localized within tetraspanin-rich microdomain regions at the cell surface, it also accumulates significantly in intracellular compartments such as late endosomes and lysosomes [51]. Similar to the expression of CD53, another tetraspanin, CD63 expression is a marker of asymmetric cell division linked to the endosomal compartment which plays a critical role in protein trafficking. It has been suggested that the asymmetric segregation of endosomes might provide a more general and evolutionarily conserved mechanism of asymmetric cell division [17]. A link between the endosomal compartment and mechanisms governing asymmetric cell divisions has been discovered in Drosophila [47]. CD63 has been implicated in maintaining hematopoietic stem cell dormancy through functional interplay with TGF-β signaling [52].

In mammals, Numb binds to α-adaptin, a crucial subunit of the AP-2 complex, thereby linking the protein to clathrin-mediated endocytic machinery [53]. Notch signaling plays a central role in T cell lineage development [54,55] and concurrently inhibits B cell differentiation [56,57], suggesting that activation of Notch-1 signaling in multipotential hemopoietic progenitors controls T vs. B cell lineage determination. During asymmetric division of thymocytes, Numb, localized to endosomal compartments, has been proposed to directly interact with Notch1, functioning as a negative regulator that constrains its signaling activity [58]. The hexokinase 2 (HK2) mitochondrial metabolic enzyme is known to localize in the nucleus from leukemic and normal hematopoietic stem cells. Overexpression of nuclear HK2 increases leukemic stem cell properties and decreases differentiation, whereas selective nuclear HK2 knockdown promotes differentiation and decreases stem cell function. In AML leukemic stem cells, HK2 interacts with DNA damage response proteins and overexpression of nuclear HK2 decreases the level of double-strand DNA breaks and increases chemo-resistance [27].

During the chronic phase of CML, after single-cell trajectory reconstitution on prognosis phenotype, CD63 regulation highlighted a trajectory cluster implicating HSPB1, PIM2, ANXA5, LAMTOR1, CFL1, CD52, RAD52, MEIS1, and PDIA3 molecules characterizing hematopoietic malignancies like myelodysplasia, acute myeloid leukemia, and acute lymphoid leukemia. HSPB1, also known as HSP27 (heat shock protein 27), is a molecular chaperone that has been found to be highly expressed in bone marrow mononuclear cells from patients newly diagnosed with AML-M4/M5. Experimental knockdown of HSP27 has been shown to enhance leukemic cell sensitivity to chemotherapy and amplify drug-induced apoptosis. [59]. Pim2 is a pro-survival kinase which has been postulated as a therapeutic target for eradication of chronic myeloid leukemia stem cells. In CML leukemic stem cells, PIM2 expression is promoted by both a BCR::ABL-dependent (IM-sensitive) Signal Transducer and Activator of Transcription 5 (STAT5)-mediated pathway and a BCR::ABL-independent (IM-resistant) Signal Transducer and Activator of Transcription 4 (STAT4)-mediated pathway [60]. ANXA5 overexpression in B cell acute lymphoblastic leukemia is implicated in glucocorticoid resistance [61]. LAMTOR1 is a member of Ragulator-Rag GTPase complex, which may provide a platform for nutrient sensing on lysosomes [62] and has been shown in vitro to be regulated by epigenetic treatment in acute promyelocytic leukemia [63]. The cofilin 1 (CFL1) signaling pathway was shown to be involved in diallyl disulfide (DADS)-induced differentiation and inhibitory effects on the proliferation, migration, and invasion of human leukemia HL-60 cells [64]. CD52 has been previously reported as upregulated during the blast crisis phase of chronic myeloid leukemia (CML) [65]. The monoclonal antibody alemtuzumab, targeting CD52, is routinely employed for T cell depletion in the context of allogeneic hematopoietic stem cell transplantation [66]. Additionally, it has demonstrated therapeutic efficacy in various hematologic malignancies, including acute lymphoblastic leukemia (ALL) [67], and in cases of chronic lymphocytic leukemia (CLL) that are relapsed or refractory to standard treatment [68]. Concerning RAD52, during CML, BCR::ABL-mediated stimulation of single-strand annealing was accompanied by enhanced nuclear colocalization of RAD52 and ERCC excision repair 1 and the endonuclease non-catalytic subunit (ERCC1), which play a key role in DNA repair [69]. MEIS1 is a major hematopoietic transcription factor implicated in metabolism because MEIS1 can sufficiently transactivate hypoxia-inducible factor 1 subunit alpha (Hif-1α) to precisely regulate glycolysis in both murine bone marrow HSCs and human-mobilized peripheral blood HSCs [70]. In AML, MEIS1 cooperates with PBX3 to drive a core transcriptome of Mixed Lineage Leukemia (MLL)-rearranged disease [71]. In acute myeloid leukemia (AML) cells, PDIA3 plays a regulatory role in key cellular processes including apoptosis, proliferation, invasion, and migration. These effects are mediated through its influence on oxidative phosphorylation, amino sugar, nucleotide sugar metabolism, and modulation of the mitogen-activated protein kinases (MAPK) signaling pathway [72].

## 5. Conclusions

This study revealed deregulation of asymmetric stem cell division markers during chronic myeloid leukemia (CML) progression, with pronounced downregulation of five key genes—CD63, SELL, NUMB, HK2, and LAMP2—during blast crisis compared to the chronic phase. These findings were corroborated by single-cell transcriptomic analysis. Trajectory reconstruction in CD34^+^CD38^−^ cells during the chronic phase further identified significant modulation of CD63, associating its expression with a leukemic transcriptional signature and poor response to tyrosine kinase inhibitor (TKI) therapy. Taken together, these results support prospective evaluation of CD63 expression in CML patients, both at diagnosis and throughout TKI treatment, as a potential prognostic biomarker.

## Data Availability

All R scripts used in the analyses in the article are available at the following web address: https://github.com/cdesterke/CD63_CML (accessed on 13 May 2025).

## Supplementary Materials

The following supporting information can be downloaded at https://www.mdpi.com/article/10.3390/bioengineering12080830/s1, Supplementary Figures: Supplementary Figure S1: Machine learning predictive sores obtained with asymmetric hematopoietic stem cell-related genes; Supplementary Figure S2: CD63 cluster genes are implicated in negative regulation of apoptotic process; Supplementary tables: Supplementary Table S1: Genes associated with asymmetric hematopoietic stem cell literature; Supplementary Table S2: Concordance table of training and validation cohorts; Supplementary Table S3: Most significant genes found in single-cell CML chronic phase prognosis trajectory.

## Abbreviations

The following abbreviations are used in this manuscript:

BCR::ABL	Breakpoint cluster region::Abelson murine leukemia viral oncogene homolog 1
CML	Chronic myeloid leukemia
CP	Chronic phase
BC	Blast crisis
ACD	Asymmetric cell divisions
MDS	Myelodysplastic syndromes
AML	Acute myeloid leukemia
ALL	Acute lymphoid leukemia
GLUT1	*alias* “SLC2A1” for Solute Carrier Family 2 Member 1
TKI	Tyrosine kinase inhibitor
PPARG	Peroxisome proliferator activated receptor gamma
PPARGC1A	PPARG coactivator 1 alpha
TEK	TEK receptor tyrosine kinase
DNML1	Dynamin-1-like protein
CD71	*Alias* “TFRC”: transferrin receptor
CDC42	Cell division cycle 42
CD34	Cluster of differentiation 34
CD38	Cluster of differentiation 38
TP53	Tumor protein p53
MYC	MYC proto-oncogene, bHLH transcription factor
TCF7L2	Transcription factor 7 like 2
SELL	Selectin L
WNT	Wingless-related Integration Site
CD63	Cluster of differentiation 63
NUMB	NUMB endocytic adaptor protein
HK2	Hexokinase 2
LAMP2	Lsosomal associated membrane protein 2
RAD51C	RAD51 paralog C
CD53	Cluster of differentiation 53
HSPB1	Heat shock protein family B (small) member 1
HSP27	Heat Shock Protein 27
PIM2	Pim-2 proto-oncogene, serine/threonine kinase
ANXA5	Annexin A5
LAMTOR1	Late endosomal/lysosomal adaptor, MAPK and MTOR activator 1
CFL1	Cofilin 1
CD52	Cluster of differentiation 52
RAD52	RAD52 homolog, DNA repair protein
ERCC1	ERCC excision repair 1, endonuclease non-catalytic subunit
MEIS1	Meis homeobox 1
PDIA3	Protein disulfide isomerase family A member 3
PBX3	PBX homeobox 3
ENO1	Enolase 1
MTRNR2L4	MT-RNR2 like 4 (pseudogene)
MTRNR2L3	MT-RNR2 like 3 (pseudogene)
HIF1α	Hypoxia inducible factor 1 subunit alpha
MAPK	Mitogen-activated protein kinases
TSC22D1	TSC22 domain family member 1
TMBIM6	Transmembrane BAX inhibitor motif containing 6
STAT5	Signal Transducer and Activator of Transcription 5
STAT4	Signal Transducer and Activator of Transcription 4
ATAD3A	ATPase family AAA domain containing 3A
MFN2	Mitofusin 2
PINK1	PTEN induced kinase 1
JAM3	Junctional adhesion molecule 3
SEPTIN6	Septin 6
PROM1	Prominin 1
SIRT7	Sirtuin 7
PRDM16	PR/SET domain 16
SEPTIN 2	Septin 2
AP2A2	Adaptor related protein complex 2 subunit alpha 2
ID1	Inhibitor of DNA binding 1, HLH protein
NR4A1	Nuclear receptor subfamily 4 group A member 1
S100A10	S100 calcium binding protein A10
FTL	Ferritin light chain
MLL	Mixed Lineage Leukemia
CLL	Chronic lymphocytic leukemia
Notch1	NOTCH receptor 1
