# Supplementary material for: Regulation of Tetraspanin CD63 in Chronic Myeloid Leukemia (CML): Single-Cell Analysis of Asymmetric Hematopoietic Stem Cell Division Genes
Source: Bioengineering (Basel). 2025 Jul 31;12(8):830. doi: 10.3390/bioengineering12080830 (PMC12383603; doi:10.3390/bioengineering12080830)
Supplement: Supplementary file 1 [file bioengineering-12-00830-s001.zip › bioengineering-3768686-supplementary.pdf]

bioengineering

MDPI

Type of the Paper (Article)

Regulation of the tetraspanin CD63 in Chronic Myeloid Leukemia (CML): Single cell analysis of asymmetric hematopoietic stem cell (HSC) division

Christophe Desterke [1], Annelise Bennaceur-Griscelli [2] and Ali G Turhan [3,*]

1 University Paris Saclay, Faculty of Medicine, INSERM UMRS-1310, Villejuif, France; christophe.desterke@inserm.fr
2 University Paris Saclay, Faculty of Medicine, INSERM UMRS-1310, Villejuif, France, INGESTEM National iPSC Infrastructure, 94800 Villejuif, France, CITHERA, Centre for IPSC Therapies, INSERM UMS-45, Genopole Campus, 91100 Evry, France ; abenna@ehotmail.fr
3 University Paris Saclay, Faculty of Medicine, INSERM UMRS-1310, Villejuif, France, INGESTEM National iPSC Infrastructure, 94800 Villejuif, France, CITHERA, Centre for IPSC Therapies, INSERM UMS-45, Genopole Campus, 91100 Evry, France ; turviv33@gmail.com

Supplemental material:

Supplemental Figures

-Supplemental Figure S1: Machine learning predictive sores obtained with asymmetric hematopoietic stell cell related genes

-Supplemental Figure S2: CD63 cluster genes are implicated in negative regulation of apoptotic process

Supplemental tables

-Supplemental Table S1: Genes associated to Asymmetric hematopoietic stem cell literature

-Supplemental Table S2: Concordance table between training and validation cohorts

-Supplemental Table S3: Top significant genes found on single cell CML chronic phase prognosis trajectory

Supplemental Figures

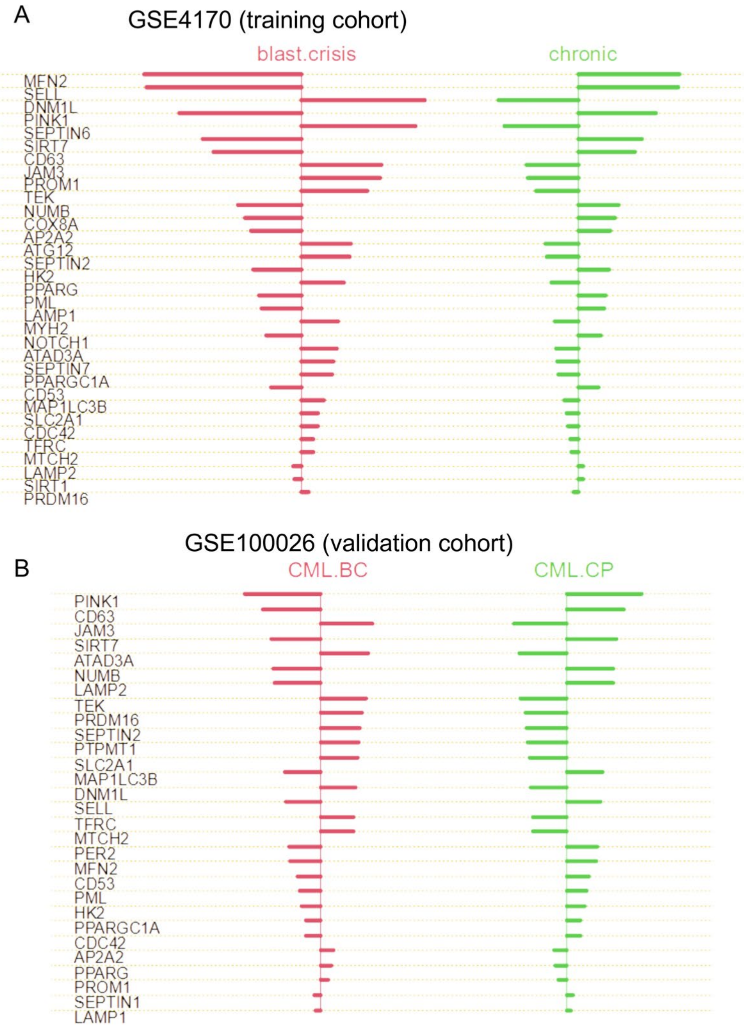

Supplemental Figure S1: Machine learning predictive sores obtained with asymmetric hematopoietic stell cell related genes: A/ Predictive scores by CML phases found on training cohort (GSE4170) for asymmetric hematopoietic stem cell related genes; B/ Predictive scores by CML phases found on validation cohort (GSE100026) for asymmetric hematopoietic stem cell related genes.

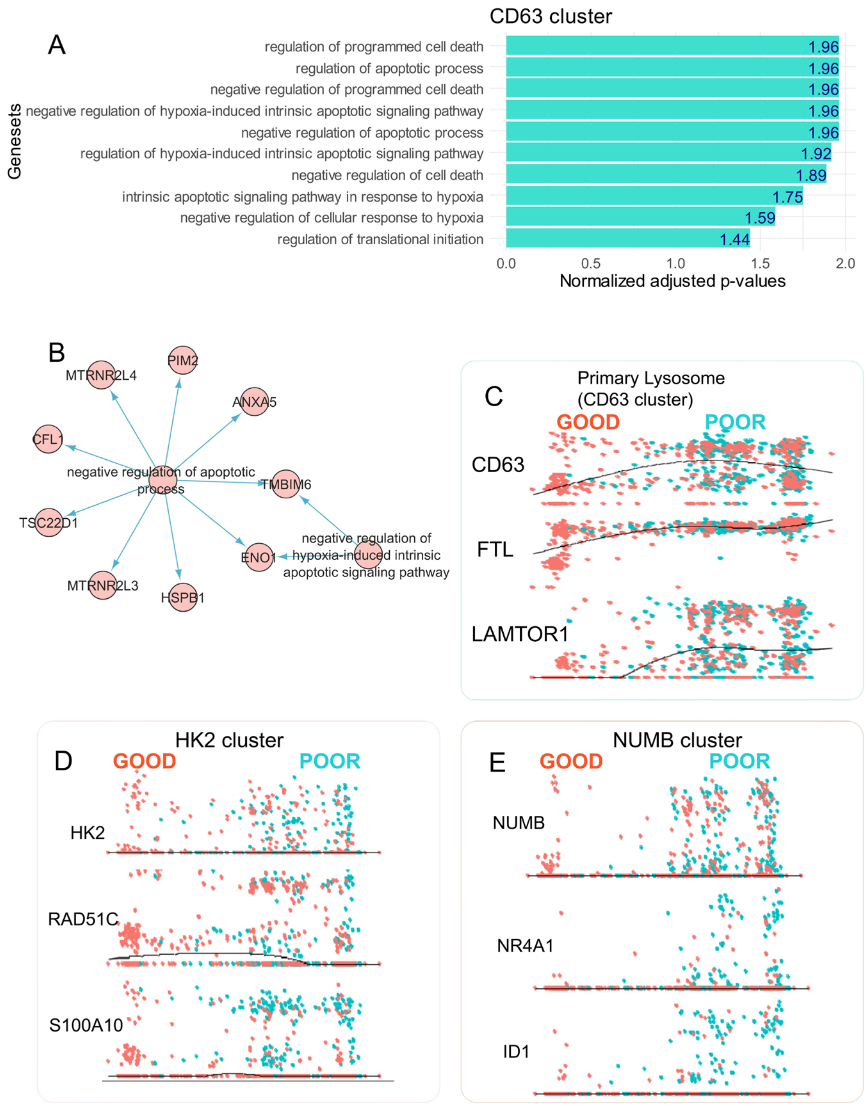

Supplemental Figure S2: CD63 cluster genes are implicated in negative regulation of apoptotic process: A/ Functional enrichment performed on Gene Ontology Biological Process database with genes from CD63 cluster; B/ Negative regulation of apoptosis process network enriched in CD63 single cell cluster; C/ Pseudotime expression of plot of markers in CD63 cluster; D/ Pseudotime expression of plot of markers in HK2 cluster

Supplemental tables

Supplemental Table S1: Genes associated to Asymmetric hematopoietic stem cell literature: columns present asymmetry HSC related genes found in literature, columns present reliability of the association, the context and the model with initial reference and Pubmed identifier of the article investigated (PMID)

human gene symbol	remark	context	model	initial reference	PMID

SEPTIN1	HSCs with enhanced mobility, decreased quiescence, increased apoptosis and defective reconstitution capacity	murine model	Ptpn21−/− HSCs	Ni et al 2019	34957123
SEPTIN6	Increased engraftment of Sept6−/− HSCs upon transplantation,	murine model	Sept6−/− HSCs	Senger et al 2017	34957123
SEPTIN7	Sept7−/− and Borg4−/− HSCs present impaired function upon transplantation and show changed Cdc42 distribution	murine model	Control HSCsSept7−/− HSCsBorg4−/− HSCs	Kandi et al 2021	34957123
BORG4	Sept7−/− and Borg4−/− HSCs present impaired function upon transplantation and show changed Cdc42 distribution	murine model	Control HSCsSept7−/− HSCsBorg4−/− HSCs	Kandi et al 2021	34957123
CDC42	Sept7−/− and Borg4−/− HSCs present impaired function upon transplantation and show changed Cdc42 distribution	murine model	Control HSCsSept7−/− HSCsBorg4−/− HSCs	Kandi et al 2021	34957123
PER2	In young HSCs (from mice that are 4 months old) there is a polar distribution of the polarity proteins	murine model	young mice	Florian et al., 2018	34957123
NUMB	In young HSCs (from mice that are 4 months old) there is a polar distribution of the polarity proteins	murine model	young mice	Florian et al., 2018	34957123
NOTCH1	organelle inheritance	NA	NA	(Loeffler et al., 2019)	34957123
CD63	organelle inheritance	NA	NA	(Loeffler et al., 2019)	34957123
DNM1L	partner of septin 2	A Drp1-mediated, distinct localization of old mitochondria is important for this asymmetric inheritance	NA	(Hinge et al., 2020)	34957123

SEPTIN2	partner of Drp1 in mitochondria	A Drp1-mediated, distinct localization of old mitochondria is important for this asymmetric inheritance	NA	(Pagliuso et al., 2016)	34957123
PML	Promyelocytic leukemia protein activates fatty acid oxidation (FAO), a metabolic mechanism required for the asymmetric divisions and maintenance of hematopoietic stem cells (HSCs).	NA	NA	(Ito et al. 2008; Ito et al. 2012;Lallemand-Breitenbach and de Thé, 2012)	34881785
PPARGC1A	PML regulates the asymmetric division and maintenance of HSCs via its involvement in the deacetylation of the PPARγ coactivator 1A (PGC1A) by SIRT1, which leads to activation of the PPAR pathway and fatty acid oxidation (FAO).	murine HSC	murine HSC	(Ito et al. 2008; Ito et al. 2012;Lallemand-Breitenbach and de Thé, 2012)	34881785
PPARG	PML regulates the asymmetric division and maintenance of HSCs via its involvement in the deacetylation of the PPARγ coactivator 1A (PGC1A) by SIRT1, which leads to activation of the PPAR pathway and fatty acid oxidation (FAO).	murine HSC	murine HSC	(Ito et al. 2008; Ito et al. 2012;Lallemand-Breitenbach and de Thé, 2012)	34881785

SIRT1	PML regulates the asymmetric division and maintenance of HSCs via its involvement in the deacetylation of the PPARγ coactivator 1A (PGC1A) by SIRT1, which leads to activation of the PPAR pathway and fatty acid oxidation (FAO).	murine HSC	murine HSC	(Ito et al. 2008; Ito et al. 2012;Lallemand-Breitenbach and de Thé, 2012)	34881785
NUMB	asymmetric cell division	segregated proteins	NA	Loeffler D Nature 2019	34059600
CD53	asymmetric cell division	segregated proteins	NA	Beckmann J Blood 2007	34059600
SELL	asymmetric cell division	segregated proteins	NA	Beckmann J Blood 2007	34059600
CD63	asymmetric cell division	segregated proteins	NA	Beckmann J Blood 2007	34059600
TFRC	asymmetric cell division	segregated proteins	NA	Beckmann J Blood 2007	34059600
AP2A2	asymmetric cell division	endocytosis HPSC	HPSC	Ting SB Blood 2012	34059600
TEK	asymmetric cell division	angiopoietin 1 receptor in PPARG FAO context	NA	Ito K Nat Med 2012	34059600
MYH2	asymmetric cell division	contractile force	NA	Shin J-WW Cell Stem Cell 2014	34059600
PROM1	asymmetric cell division	receptor stem cell marker	NA	Görgenes A Stem Cell Reports 2014	34059600
CDC42	asymmetric cell division	Aging alters epigenetics HSC asymmetry	NA	Florian MC Plos biol 2018	34059600
LAMP1	asymmetric cell division	lysosome	NA	Loeffler D Nature 2019	34059600
LAMP2	asymmetric cell division	lysosome	NA	Loeffler D Nature 2019	34059600
MAP1LC3B	autophagosome	cord blood HSC	NA	Loeffler D Nature 2019	34059600
COX8A	Cytochrome C Oxidase Subunit 8A	asymmetry segregation of damaged mitochondria during HSC division	NA	Hinge A Cell Stem Cell 2020	34059600

SLC2A1	Facilitative glucose transporter, which is responsible for constitutive or basal glucose uptake	in vivo IFNA administration increases markers asymmetry between daughter cells	in vivo	Girotra M IJMS 2020	34059600
JAM3	Junctional Adhesion Molecule 3	in vivo IFNA administration increases markers asymmetry between daughter cells	in vivo	Girotra M IJMS 2020	34059600
HK2	Hexokinase 2	in vivo IFNA administration increases markers asymmetry between daughter cells	in vivo	Girotra M IJMS 2020	34059600
NUMB	asymmetry of VSEL in cord blood	cord blood	VSEL	Ratajczak J 2011	31642043
NUMB	Numb is present on HSCs and segregates into one of the two daughter cells during the asymmetric divisions. Although Numb is not strictly required in HSCs	NA	NA	Wilson A 2007	30051749
AP2A2	Ap2a2 positively regulates HSC activity and also shows unequal segregation during mitosis are highly intriguing and indicate that Ap2a2 may in fact act as a cell fate determinant influencing ACD.	NA	NA	Ting SB, blood 2012	30051749
TEK	only top hierarchical HSCs underwent SD, in which both daughter cells are HSCs and retain Tie2 positivity	NA	NA	Ito K	29807063

PINK1	In depolarized mitochondria, the degradation of PINK1 (PTEN-induced putative kinase 1) is impaired, leading to the accumulation and activation of this kinase on the mitochondrial outer membrane	autophagy	NA	Stolz A & Dikic I 2014	29807063
MTCH2	loss of mitochondrial carrier homologue 2 (MTCH2) increases mitochondrial respiration and intracellular ROS, triggering HSC entry into the cell cycle and compromising self-renewal capacity	metabolism	NA	Maryanovich M et al Nat Com 2015	29807063
SIRT7	deletion of Sirtuin 7 (Sirt7) increases mitochondrial unfolded protein stress, as well as mitochondrial biogenesis and respiration, leading to impaired regenerative capacity with a loss of quiescence and a shift in metabolic process that signals cellular differentiation.	mitochondria	NA	Mohrin M et al. Science 2015	29807063
PTPMT1	disrupting mitochondrial Oxphos upon the loss of Ptpmt1, a mitochondrial phosphatase targeting phosphatidylinositol phosphates, blocks early HSC differentiation and results in rapid hematopoietic failure in vivo.	mitochondrial oxphos	in vivo	Yu WM et al. Cell Stem Cell 2013	29807063
ATG12	Defective autophagy by the ablation of Atg12 accelerates blood aging phenotypes, with myeloid-biased lineage distribution and elevated Oxphos	autophagy	NA	Ho TT et al. Nature 2017	29807063

ATAD3A	Conditional deletion of Atad3a in adult hematopoietic cells leads to the accumulation of Pink1 and the enhancement of mitophagy. Atad3a conditional knockout mice exhibited blocked hematopoietic lineage commitment at the progenitor stage, and enlarged HSPC pools.	mitophagy	murine	Jin G et al Nature immunology 2018	29807063
PRDM16	Prdm16 (PR domain containing 16) - Mitofusin-2 (Mfn2) axis contributes to the maintenance of HSCs with lymphoid potential by buffering calcium levels through mitochondrial tethering to the endoplasmic reticulum	mitochondria	NA	Aguilo F et al blood 2011	29807063
MFN2	Prdm16 (PR domain containing 16) - Mitofusin-2 (Mfn2) axis contributes to the maintenance of HSCs with lymphoid potential by buffering calcium levels through mitochondrial tethering to the endoplasmic reticulum	mitochondria	NA	Luchsinger LL Nature 2016	29807063

Supplemental Table S2: Concordance table between training and validation cohorts: for each asymmetric HSC marker machine learning blast crisis (BC) predictive score were presented in training cohort (train.BC) and in validation cohort (valid.BC), in last column concordance of regulation in the two bulk transcriptome cohorts is describe.

gene symbol	train.BC	valid.BC	concordance
PINK1	-0.8799	-0.6958	negative_YES
SELL	-1.123	-0.3571	negative_YES
MFN2	-1.1381	-0.325	negative_YES
DNM1L	0.8879	0.3683	positive_YES
SIRT7	-0.7093	-0.4832	negative_YES
CD63	-0.6326	-0.5485	negative_YES
JAM3	0.5803	0.5044	positive_YES
TEK	0.4779	0.4505	positive_YES
NUMB	-0.4571	-0.4622	negative_YES
SEPTIN6	0.8262	-0.0092	other
SEPTIN2	0.3477	0.3992	positive_YES
ATAD3A	0.2522	0.4657	positive_YES
PROM1	0.5678	0.1442	positive_YES
HK2	-0.3445	-0.2279	negative_YES
PML	-0.3023	-0.2493	negative_YES
AP2A2	-0.3619	0.1809	other
MAP1LC3B	0.1596	-0.3725	other
LAMP2	-0.0572	-0.4576	negative_YES
SLC2A1	0.1233	0.3787	positive_YES
CD53	-0.2235	-0.2646	negative_YES
PPARG	0.3035	0.1676	positive_YES
PRDM16	0.0525	0.4134	positive_YES
COX8A	-0.4074	-0.0433	negative_YES
TFRC	0.088	0.3497	positive_YES
ATG12	0.3605	0.0768	positive_YES
MTCH2	0.0824	0.3479	positive_YES
PPARGC1A	0.2247	-0.1981	other
LAMP1	-0.2917	-0.1188	negative_YES
CDC42	0.1195	-0.1925	other
SEPTIN7	0.2303	0.0692	positive_YES
NOTCH1	-0.2533	0.0174	other
MYH2	0.2614	0	other
SIRT1	-0.0527	-0.0269	negative_YES

Supplemental Table S3: Top significant genes found on single cell CML chronic phase prognosis trajectory: columns present the False Discovery Rate adjusted p-value of gene single cell expression associated to the pseudotime cell trajectory, number of cells expression the molecule and fraction of cells expressing it.

gene symbol	Trajectory q-value	num_cells_expressed	fraction expressed
RPL32	9,18E-70	388	52,08
CLEC4GP1	2,77E-38	724	97,18
TSC22D3	2,56E-33	680	91,28
TTC34	9,16E-25	288	38,66
MMEL1	3,99E-23	359	48,19
PRSS21	7,11E-16	217	29,13
HNRNPL	3,22E-14	743	99,73
AVP	4,07E-14	270	36,24
ANXA5	1,34E-13	428	57,45
RPS25	7,19E-13	745	100,00
CYTIP	2,34E-12	296	39,73
LRRC75A-AS1	1,30E-11	745	100,00
SH3BGRL3	2,38E-10	718	96,38
ID1	4,63E-10	135	18,12
OXT	1,52E-09	226	30,34
FTL	1,54E-09	741	99,46
MTRNR2L3	1,97E-09	744	99,87
ANKRD28	2,53E-09	586	78,66
PPP1R15A	2,50E-08	448	60,13
S100A10	1,03E-07	274	36,78
PDIA3	1,03E-07	647	86,85
PIM2	1,32E-07	435	58,39
CD52	1,87E-07	640	85,91
DUSP1	2,67E-07	671	90,07
TBCB	2,67E-07	394	52,89
IGLL1	3,56E-07	296	39,73
FTH1P3	4,28E-07	626	84,03
AREG	6,28E-07	200	26,85
SLC25A19	7,61E-07	218	29,26
HLA-H	2,62E-06	654	87,79
ANXA1	3,12E-06	742	99,60
PFN1	4,08E-06	694	93,15
TSC22D1	4,08E-06	692	92,89
ANXA2P1	6,20E-06	237	31,81
TSTD1	6,20E-06	561	75,30
ECH1	6,46E-06	731	98,12
ANXA2P2	6,73E-06	493	66,17
HLA-E	7,07E-06	726	97,45
HBB	7,55E-06	134	17,99
MEIS1	7,97E-06	690	92,62
AMICA1	8,17E-06	348	46,71
MED12L	9,34E-06	451	60,54
PDIA3P1	1,08E-05	508	68,19

ARPC1B	1,12E-05	636	85,37
CFL1	1,42E-05	727	97,58
ANXA2P3	1,44E-05	225	30,20
RNF130	1,94E-05	479	64,30
RPL23	1,94E-05	745	100,00
TAGLN2	1,94E-05	686	92,08
MTRNR2L8	2,37E-05	745	100,00
TMBIM6	2,84E-05	729	97,85
RPS11	2,86E-05	745	100,00
MBOAT1	2,97E-05	743	99,73
RPL18A	3,13E-05	745	100,00
ANXA2	3,28E-05	487	65,37
MTRNR2L4	3,28E-05	735	98,66
GUCY1A3	3,40E-05	509	68,32
RAD52	3,40E-05	501	67,25
SERF2	3,90E-05	742	99,60
ADAM19	3,93E-05	145	19,46
NR4A1	3,93E-05	110	14,77
HLA-F	4,06E-05	433	58,12
MXRA8	4,91E-05	561	75,30
IGJ	4,94E-05	215	28,86
MMRN1	5,09E-05	552	74,09
CD63	5,63E-05	660	88,59
RAD51C	6,06E-05	303	40,67
PIP4K2A	6,32E-05	663	88,99
HSPB1	6,53E-05	629	84,43
LAMTOR1	6,74E-05	468	62,82
HNRNPH1	7,45E-05	745	100,00
PPP1CA	7,53E-05	669	89,80
EIF4G2	8,49E-05	717	96,24
ENO1	8,49E-05	739	99,19
RPL27	1,03E-04	745	100,00
